# The Effect of Self-Management on Patients with Chronic Diseases: A Systematic Review and Meta-Analysis

**DOI:** 10.3390/healthcare12212151

**Published:** 2024-10-29

**Authors:** Yanfang Huang, Sijia Li, Xiuli Lu, Weiqiang Chen, Yun Zhang

**Affiliations:** 1 School of Nursing, Guangdong Pharmaceutical University, Guangzhou 510006, China; 2 School of Basic Medical Sciences, Guangdong Pharmaceutical University, Guangzhou 510006, China

**Keywords:** self-management, chronic diseases, meta-analysis, quality of life, self-efficacy, depression, anxiety

## Abstract

Background: Chronic diseases significantly impact global morbidity and mortality, affecting millions. Self-management interventions are crucial for improving patient health outcomes. This study explores the effects of self-management interventions on the quality of life (QOL), self-efficacy, depression, and anxiety of patients with chronic diseases. Methods: Relevant studies were searched from PubMed, EMBASE, and Web of Science. Two reviewers independently screened the literature, evaluated the risk of bias assessment, and extracted characteristics and outcomes among patients with chronic diseases. For each included study, we calculated the standardized mean difference (SMD) and 95% confidence interval (CI) of the main outcomes. When deemed feasible, the heterogeneity of the study was explored by meta-analysis and subgroup analysis. Results: Thirty-four studies involving a total of 7603 patients with chronic diseases were included. Self-management interventions significantly improved quality of life (Higher-better QOL and Lower-better QOL), self-efficacy, and reduced depression symptoms compared to usual care (95%CI 0.01 to 0.15, *p* = 0.03; 95%CI −0.49 to −0.08, *p* = 0.006; 95%CI 0.19 to 0.62, *p* < 0.001; 95%CI −0.23 to −0.07, *p* < 0.001). However, no significant effect was found for anxiety (95%CI −0.18 to 0.03, *p* = 0.18). In the heterogeneity analysis, Lower-better QOL and self-efficacy were all higher than 50% (I^2^ = 80%, 87%). After the subgroup analysis, the heterogeneity of Lower-better QOL and self-efficacy was less than 50% (I^2^ = 0%, 16.1%). Subgroup analyses revealed that studies with mean age greater than 60 years old and follow-up times greater than 6 months were more effective in improving patients’ Lower-better QOL (*p* = 0.03, *p* = 0.004), whereas follow-up times less than 6 months were better at reducing patients’ anxiety symptoms (*p* = 0.03). Conclusions: Self-management interventions are more effective than routine care in managing chronic diseases, significantly improving patients’ quality of life, self-efficacy, and reducing depressive symptoms, but they did not show significant improvements in anxiety symptoms. Overall, self-management interventions for chronic diseases can help patients adapt to the changes brought about by the disease and self-manage diseases to prevent disease progression.

## 1. Introduction

The continued acceleration of human aging will create a global burden, such as the rising proportion of unhealthy older people [1]. Aging is a major risk factor for chronic fatal diseases [2]. As longevity increases, an individual’s health declines and the probability of developing various chronic diseases increases. Chronic diseases mainly include cardiovascular diseases (CVDs), cancer, diabetes, and chronic respiratory diseases (CRDs), also known as noncommunicable diseases (NCDs). NCDs are the leading global cause of death and are responsible for just over 70% of deaths worldwide [3]. CRD and CVD have a particularly significant impact on older adults. For example, in 2017, an estimated 3.91 million people died from chronic respiratory diseases, of which, 3.2 million (81.7%) died from chronic obstructive pulmonary disease (COPD) [4]. Similarly, cardiovascular diseases are a major cause of high mortality rates among older adults. According to a WHO report, in 2019, cardiovascular disease deaths accounted for 33% of all global deaths. Moreover, 80% of CVD deaths can be prevented through optimized risk factor management, including smoking cessation and increased physical activity [5]. The key risk factors underlying chronic diseases are hypertension, obesity, physical inactivity, tobacco use, harmful alcohol consumption, and trans fatty acids (TFAs) intake [6]. Due to the long course of chronic diseases and the delay of the diseases, the quality of life and productivity of patients can be negatively affected. If not effectively managed, it can lead to acute and long-term complications requiring expensive hospitalizations and readmissions [7].

Healthy lifestyles such as personalized healthy eating patterns and increased physical activity can promote patient health and reduce the duration and severity of illness [8,9]. Self-management is defined as the day-to-day management of chronic conditions by individuals throughout an illness [10]. The term “self-management” indicates that patients with chronic diseases manage their disease as it progresses [11]. It suggests that patients with chronic diseases are the ones who manage their illnesses, as only they themselves can be continuously responsible for the daily care of their diseases. Traditional healthcare models have consistently focused on managing specific disease conditions rather than providing comprehensive patient care, proving to be both costly and ineffective in the treatment of chronic diseases. Given that many patients face multiple chronic illnesses alongside challenging living environments, this approach hinders patients’ ability to effectively self-manage their conditions. Studies show that a significant portion of unnecessary healthcare expenditures and poor health outcomes associated with chronic disease treatment primarily stem from patients’ inability to effectively self-manage their conditions. Patients with chronic diseases who engage in disease self-management do have a better disease prognosis [12]. There are many forms of self-management of chronic diseases, such as self-management based on networks, telephone, meetings, printed materials, etc. Self-management of chronic diseases can improve patients’ clinical indicators, such as systolic blood pressure, diastolic blood pressure, and HbA1c [13]. Meanwhile, it can improve patients’ depression and anxiety [14]. Meta-analysis evidence demonstrates that self-management interventions have positive impacts on patients with various chronic conditions, including, but not limited to, asthma, chronic heart failure, chronic obstructive pulmonary disease, and type 2 diabetes mellitus [15]. With the extension of the self-management cycle, patients will change their behavior to improve their quality of life and reduce complications. Effective combinations of prevention, treatment, and education are encouraged to educate patients to strengthen self-management, improve living habits, prevent disease deterioration, and ultimately control the overall medical cost [16].

The results of existing randomized controlled studies are inconsistent due to the different combinations and means of implementation of self-management interventions for chronic diseases [17,18,19,20,21]. In addition, several studies have systematically reviewed and meta-analyzed the impact of self-management interventions on clinical indicators in patients with chronic diseases. In a systemic review and meta-analysis of self-management of hypertension using mobile health, it was concluded that mHealth interventions resulted in better blood pressure control [22]. Type 2 diabetes mellitus (T2DM) self-management via mHealth app could reduce T2DM patients’ hemoglobin A1c (HbA1c) [23,24]. However, these studies have focused primarily on specific clinical indicators, and there have been relatively few systematic assessments of the impact of nonclinical indicators such as quality of life, self-efficacy, depression, and anxiety in patients with chronic diseases. Therefore, this study aims to explore the effects of self-management interventions on the quality of life, self-efficacy, depression, and anxiety of patients with chronic diseases through meta-analysis. This study identified the advantages of self-management interventions through meta-analysis to provide a theoretical basis for future self-management interventions in chronic diseases.

## 2. Methods

This systematic review was conducted in accordance with the PRISMA 2020 guidelines [25]. The protocol has been previously registered on PROSPERO, registration number CRD42022319681. This systematic review and meta-analysis were reported in line with the Preferred Reporting Items for Systematic reviews and Meta-Analyses (PRISMA) statement guidelines and are shown in the Supplementary Materials.

### 2.1. Eligibility Criteria and Study Search

This systematic review included a randomized controlled trial (RCT). The included studies met the following criteria: (1) randomized controlled trial design; (2) patients have one or more chronic diseases (the diseases meet the diagnostic criteria); (3) patients are over 18 years of age; (4) patients themselves are involved in self-management intervention. The exclusion criteria are as follows: (1) the studies without complete data or lack of the main outcome measures were excluded; (2) studies still in the intervention period were excluded; (3) cancer and psychiatric patients were also excluded because the disease progression affects the related outcomes; (4) literature for which the full text could not be obtained; (5) duplicate publication of literature.

We searched PubMed, EMBASE, and Web of Science for the effect of self-management on patients with chronic diseases. The studies we searched were published between January 2016 and December 2021. The search was conducted using the following keywords: “chronic diseases”, “heart disease”, “hypertension”, “stroke”, “Parkinson’s disease”, “obesity”, “diabetes”, “COPD”, “intestinal disease”, “chronic renal diseases”, “self-management”, and so on. Relevant variations of search terms were obtained from database thesauri and pertinent review articles. The detailed search formulas are shown in the Supplementary Materials.

The two researchers independently evaluated the abstracts and full text of the articles, and included the articles that met the eligibility criteria in the meta-analysis. For full-text articles, disagreements were resolved through discussion with a third researcher, who provided an independent assessment to reach a consensus.

### 2.2. Outcomes

With the changes in medical models, in addition to the need to pay attention to changes in patient clinical indicators, people are increasingly paying attention to the quality of survival and psychological issues of chronic disease patients. So, the main outcomes were quality of life (QOL), self-efficacy, depression, and anxiety in this study. The results of the included study were measured by different scales. Due to the different criteria for judging the scores of the quality of life scale, the higher the scores of some of the quality of life scales, the better the quality of life, so we referred to this section collectively as Higher-better QOL. The opposite is true for the other part of the scale, which is called Lower-better QOL. The former was divided into Higher-better QOL [EuroQol Five Dimensions Questionnaire(EQ-5D), Short Form Six Dimension (SF-6D), the 18-item Korean version of Kidney Disease Quality of Life Instrument-Short Form (KDQOL), The Mini-Asthma Quality of Life Questionnaire (MAQOL), and Quality of Life in Epilepsy (QOLIE-31P)] [17,18,19,20,26,27,28,29,30,31,32,33] and the latter into Lower-better QOL [The Minnesota Living with Heart Failure Questionnaire (MLHFQ), The COPD-Assessment Test (CAT), The St. George Respiratory Questionnaire (SGRQ), and The Impact of Weight on Quality of Life-Lite] [17,18,34,35,36,37,38,39,40,41,42,43].

### 2.3. Data Extraction

Two authors independently completed data extraction and the data were recorded on the Microsoft Word table we created. Extracted data included first author, year of publication, country, type of chronic diseases, sample size, mean age, the proportion of men, follow-up time, primary outcomes, definition of intervention, and definition of control. In addition, if data for the primary outcomes were lacking, this study obtained the corresponding data by calculating the relevant formulae whenever possible.

### 2.4. Risk of Bias Assessment

Two reviewers independently used the Cochrane Collaboration tool to assess the methodological quality and reliability of the trial for each included study [44]. The following risk of bias items were evaluated: random sequence generation (selection bias), allocation concealment (selection bias), blinding of participants and personnel (performance bias), blinding of outcome assessment (detection bias), incomplete outcome data (attrition bias), selective reporting (reporting bias), and other bias.

### 2.5. Statistical Analysis

The statistics were inputted by two authors independently. Review Manager 5.3 software and StataSE 12.0 were used to merge the research data for meta-analysis. For each included study, we calculated the standardized mean difference (SMD) of the main outcomes. The pooled SMD and 95% confidence interval (CI) of the primary outcome measures were calculated using the inverse-variance random-effects model. The Cochrane Q test was used to calculate I^2^ to detect the consistency of the included studies [45]. If I^2^ is greater than 50%, it indicates that there is substantial heterogeneity. This study evaluated the effect of studies on random effects model heterogeneity by removing one study at a time. Then, we decided to conduct a subgroup analysis according to different measurement scales of the main results, to explore the heterogeneity of the studies. Moreover, publication bias was tested using Begg’s test and trim-and-fill analysis.

## 3. Results

### 3.1. Selection of Studies

**Figure 1:**
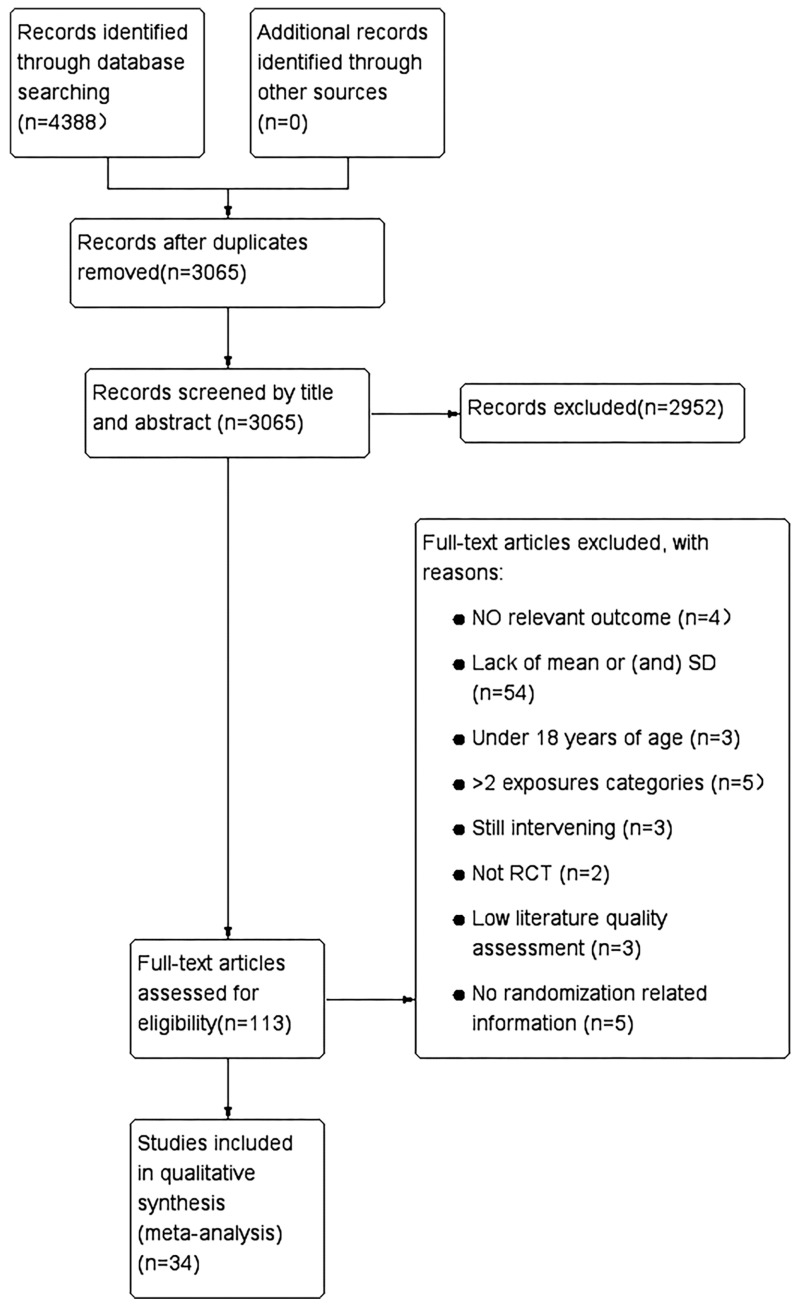
Flow chart of study selection.

A total of 4388 articles were searched according to the search strategy. We removed 1323 articles through duplicate checking, and 3065 articles remained. A total of 2952 articles were excluded by screening the topics and abstracts of the studies. The eligibility of the remaining 113 full-text articles were assessed, leaving 34 articles. A total of 79 articles were excluded for the following reasons: no relevant outcomes, lack of related data, under 18 years of age, >2 exposure categories (only studies with only 2 subgroups, i.e., self-management and usual care, were included in this article; articles with 3 or more subgroups were excluded), still intervening (it was not clear from the title and abstract that this article was an ongoing intervention study), low literature quality assessment (the quality of the literature was assessed using the Cochrane risk of bias scale, and each entry showed “high risk”), and no randomization-related information (the article was shown to be an RCT study, but the randomization method was not specified in the methodology). The complete process of studies screening is shown in Figure 1.

### 3.2. Study and Patient Characteristics

A total of 7603 patients with chronic diseases were included in this systematic review. The diseases included in the studies were summarized as follows: thirteen studies (38.24%) were CRD [18,27,29,30,31,34,37,38,39,40,42,46,47], including COPD, chronic asthma, and bronchiectasis; ten studies (29.41%) were CVD [17,26,28,32,33,35,36,41,48,49], including chronic heart failure (CHF), stroke or TIA, hypertension, epilepsy, and coronary heart disease (CHD); four studies (11.76%) were chronic kidney diseases [19,21,50,51], including pre-end-stage renal disease and end-stage renal disease (ESRD); five studies (14.71%) were diabetes [52,53,54,55,56]; one study (2.94%) was multimorbidity [20]; one study (2.94%) was obesity [43]. The mean age was between 41.7 and 71.6 years old. The proportion of men was between 25% and 89.1%. Follow-up times ranged from 2 weeks to 24 months. Forms of the self-management invention were as follows: Health coaching, Group meeting, Based on Internet and mHealth technologies, Group meeting and Health coaching, Group meeting and Family visit, Based on Internet and mHealth technologies and Health coaching, Printed materials and Health coaching, Group meeting and Health coaching/Family visit and Group meeting and Based on Internet and mHealth technologies. The main characteristics of the studies included in the systematic review are shown in Table S1 (available as Supplementary Materials). The characteristics of the self-management intervention in each study are shown in Table S2 (available as Supplementary Materials).

### 3.3. Risk of Bias Assessment of Included Studies

**Figure 2:**
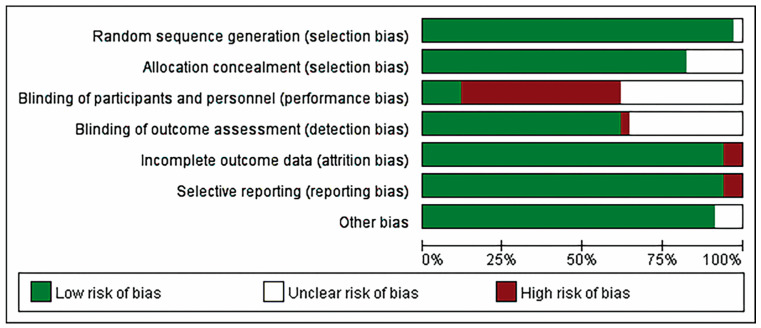
Risk of bias graph: review authors’ judgements about each risk of bias item presented as percentages across all included studies.

Due to the nature of the intervention, RCTs are difficult to do double-blind. In the articles included in this study, nearly half of the participants and the main researchers were not blinded, and the statisticians were blinded, but this did not lead to bias. In addition to blinding of participants and personnel (performance bias), most other risks of items were low risk or unclear risk. In the blinding of participants and personnel (performance bias) item, 50% of studies showed high risk (Figure 2).

### 3.4. Quality of Life

**Figure 3:**
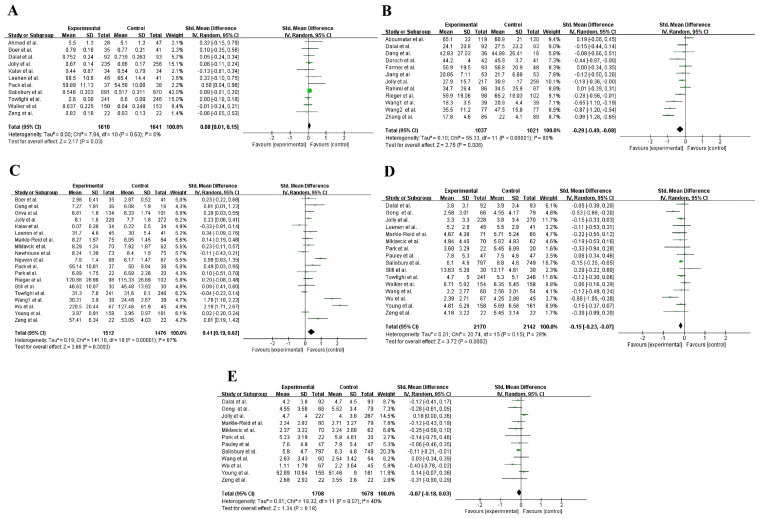
Forest plot: comparison of the effects of self-management and usual care on patients with chronic diseases. (**A**) Higher-better QOL [17,18,19,20,26,27,28,30,31,32,33]; (**B**) Lower-better QOL [17,18,34,35,36,37,38,39,40,41,42,43]; (**C**) Self-efficacy [18,19,21,26,27,28,32,33,36,38,43,46,47,50,51,52,53,54,55]; (**D**) Depression [17,18,20,21,26,30,32,33,40,47,48,52,53,54,55,56]; (**E**) Anxiety [17,18,20,21,32,40,47,52,53,54,55,56].

The difference in Higher-better QOL and Lower-better QOL between usual care and self-management interventions of patients with chronic diseases were both statistically significant (SMD 0.08, 95%CI 0.01 to 0.15, *p* = 0.03; SMD −0.29, 95%CI −0.49 to −0.08, *p* = 0.006) (Figure 3A,B). It is shown that self-management interventions can improve patients’ quality of life.

### 3.5. Self-Efficacy

There was a statistically significant difference in self-efficacy between routine nursing and self-management interventions of patients with chronic diseases (SMD 0.41, 95%CI 0.19 to 0.62, *p* < 0.001) (Figure 3C). It is shown that self-management interventions can improve patients’ self-efficacy.

### 3.6. Depression

There was a statistically significant difference in depression between routine nursing and self-management interventions of patients with chronic diseases (SMD −0.15, 95%CI −0.23 to −0.07, *p* < 0.001) (Figure 3D). Self-management interventions are beneficial in reducing patients’ depressive symptoms.

### 3.7. Anxiety

There was no statistically significant difference in anxiety between routine nursing and self-management interventions of patients with chronic diseases (SMD −0.07, 95%CI −0.18 to 0.03, P = 0.18) (Figure 3E). This study failed to show that self-management interventions improved patients’ anxiety symptoms.

### 3.8. Heterogeneity Analysis and Sensitivity Analysis

**Figure 4:**
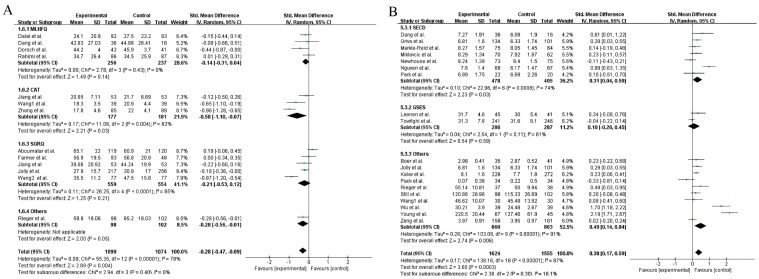
Forest plot: comparison of the effects of self-management and usual care on patients with chronic diseases using subgroup analysis. (**A**) Lower-better QOL [17,18,34,35,36,37,38,39,40,41,42,43]; (**B**) Self-efficacy [18,19,21,26,27,28,32,33,36,37,43,46,47,48,50,51,52,53,55].

**Figure 5:**
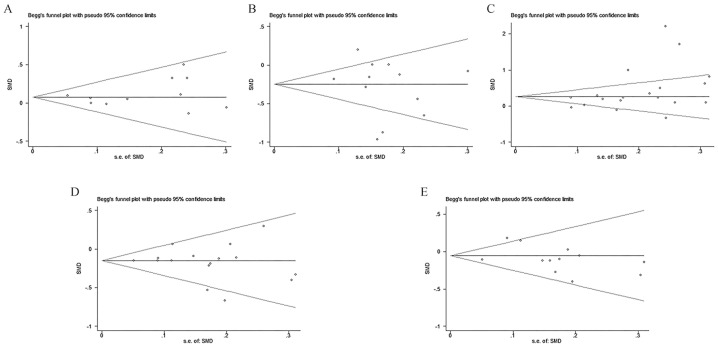
Begg’ s funnel plot: (**A**) Higher-better QOL; (**B**) Lower-better QOL; (**C**) Self-efficacy; (**D**) Depression; (**E**) Anxiety.

In the heterogeneity analysis, Higher-better QOL, depression, and anxiety had heterogeneity lower than 50% (I^2^ = 0%, 28%, and 40%) (Figure 3A,D,E), but Lower-better QOL and self-efficacy indicated high heterogeneity (I^2^ = 80%, 87%) (Figure 3B,C). To assess the stability of the main outcomes, sensitivity analysis was performed by excluding one study at a time. The results did not change significantly, indicating that the results are relatively stable. However, the I^2^ of Lower-better QOL and self-efficacy remained greater than 50%. Due to the medium to high level of heterogeneity, we decided to analyze Lower-better QOL and self-efficacy using subgroup analysis. Subgroups were performed according to the measurement scale. After the subgroup analysis, the heterogeneity of Lower-better QOL and self-efficacy was less than 50% (I^2^ = 0%, 16.1%) (Figure 4A,B). Except for self-efficacy, Begg’s test results for Higher-better QOL, Lower-better QOL, depression, and anxiety showed no publication bias (*p* > 0.05) (Figure 5A–E). In trim-and-fill analysis, it can be concluded that publication bias of self-efficacy has little effect on the results and the results are stable.

### 3.9. Subgroup Analysis

The effects of mean age, follow-up times, and forms of self-management interventions on Higher-better QOL, Lower-better QOL, self-efficacy, depression, and anxiety were analyzed by subgroup analysis, and the results are shown in Table S3 (available as Supplementary Materials). There was a statistically significant difference in the degree of improvement in Lower-better QOL in studies with mean age greater than 60 years old and follow-up times greater than 6 months compared with studies with mean age less than 60 years old and follow-up times less than 6 months. The Higher-better QOL, self-efficacy, depression, and anxiety improvement are greater in studies with the mean age more than 60 years old than in studies that are less than 60 years old. Improvements in Higher-better QOL and depression were greater in studies with greater than 6 months of follow-up times than in studies with less than 6 months. However, compared with studies with follow-up times greater than 6 months, follow-up times less than 6 months showed a larger overall effect of anxiety. Improvements in self-efficacy are less in studies with greater than 6 months of follow-up times than in studies with less than 6 months. None of the included studies were able to capture which type of self-management intervention was better at improving Higher-better QOL, Lower-better QOL, self-efficacy, depression, and anxiety.

## 4. Discussion

In this systematic review and meta-analysis, we reviewed studies published from 2016 to 2021 to assess the impact of self-management interventions on patients with chronic diseases. We found that self-management can improve the quality of life and self-efficacy of patients with chronic diseases and reduce depressive symptoms, but we did not find its positive effect on anxiety. These favorable outcome indicators are sufficient to illustrate the effectiveness of self-management in patients with chronic diseases.

The heterogeneity of Lower-better QOL and self-efficacy was all greater than 50%, so we decided to conduct subgroup analysis according to different outcome measurement scales. The results proved that it was related to the difference in outcome measurement scales. This may be due to the fact that the included studies used inconsistent survey scales, and the scoring standard as well as the total scale scores were often quite different between the scales. Furthermore, according to the subgroup analysis, mean age and follow-up times were one of the sources of heterogeneity for Low-better QOL and self-efficacy, respectively (I^2^ = 0%, Figure S1 in Supplementary Materials; I^2^ = 0%, Figure S2).

The existing RCT research results on the effect of self-management interventions on patients with chronic diseases are also inconsistent, but self-management interventions for chronic diseases are recognized to be beneficial to patients. It is important to understand the comparative advantages of these different self-management intervention models, especially as different models may be more or less amenable to wider implementation, which can maximize the effectiveness and efficiency of interventions [57].

Only some studies have demonstrated that self-management interventions improve the quality of life of patients with chronic diseases, but they did not change the beneficial impact of chronic disease self-management interventions on patients’ quality of life. Patients’ quality of life takes longer to show up in chronic disease interventions, and requires long-term follow-up to see the impact on physical outcomes [52]. The subgroup analysis of this study shows that patients over the age of 60 experienced more significant improvements in quality of life. This may be due to the fact that chronic disease patients over 60 often suffer from more severe and complex conditions, leading to lower baseline quality of life. Additionally, older adults have a stronger motivation to improve their quality of life because they are more aware of how health issues impact their daily lives. Older individuals may have more time and energy to invest in self-intervention measures, thereby enhancing adherence and ensuring the effective implementation of these interventions. Therefore, personalized health management plans should be developed for patients over 60, providing support in multiple areas such as health education, social support, psychological counseling, and social activities. Previous research has indicated that providing supportive caregiver education can potentially improve patients’ quality of life [58]. The results of this paper also showed that patients had a better quality of life in interventions with follow-up times greater than 6 months. This indicates that follow-up duration is an important factor for improving the quality of life of patients with chronic diseases. The impact of self-management interventions on patients with chronic diseases is progressive. As time extends, the benefits patients gain from self-management interventions gradually accumulate, leading to a significant improvement in their quality of life. However, the periodicity of quality of life measurements was not consistent from study to study, so some studies were unable to show the effectiveness of the interventions. In addition, patients did not recognize the importance of self-management interventions of the disease in the early stage of the disease with mild symptoms, which may lead to the occurrence of related complications, and thus expensive medical expenses [59]. The finding of a meta-analysis was consistent with this study and showed that self-management intervention can improve patients’ quality of life [12]. It has also been demonstrated that lifestyle factors play different roles in different groups, and that healthy lifestyle choice strategies can positively impact later stages of the disease, improve quality of life, and reduce healthcare utilization [60]. When educating patients about disease self-management, strategies can be chosen based on the group’s generalization of a healthy lifestyle. Therefore, we believe that self-management education can improve the awareness of chronic disease patients on disease self-management and the implementation of self-management, which can greatly improve the quality of life of patients. At the same time, healthcare institutions and policymakers should recognize the importance of long-term follow-up and establish and improve related follow-up systems (such as regular outpatient follow-ups, telephone consultations, online app platforms, and family doctor sign-ups) to ensure that patients receive continuous attention and support, thereby enhancing adherence to self-management interventions.

At the same time, this study found that self-management interventions in patients with chronic diseases have good effects on self-efficacy. This result is consistent with previous meta-analyses, further confirming the crucial role of self-management in chronic disease management [61,62]. Self-efficacy is described as efficacy expectation, which has a directive influence on the choice of activities and settings [63]. People should have exact beliefs in their behavior and thus obtain the corresponding results. Patients with chronic diseases can improve their quality of life through self-management, thus enhancing their confidence. Self-management interventions not only significantly enhance the self-efficacy and expected outcomes of cardiovascular disease survivors, but also increase their satisfaction with their self-management behavior performance, enabling them to better manage their health conditions in daily life [64]. Self-efficacy and self-management are closely related, and improving patients’ self-efficacy can also increase their self-care behaviors and improve disease control [65]. Self-efficacy can encourage patients to master disease knowledge and rehabilitation skills, improve patients’ compliance, avoid disease recurrence, delay disease progression, and reduce the incidence of complications, thereby saving medical resources and reducing the burden on patients and society [66].

With the change in the medical model, more and more attention has been paid to the psychosocial problems of patients with chronic diseases. Chronic diseases can affect patients’ mental health, with factors such as lifestyle and the number of illnesses increasing the risk of stress, anxiety, and depressive symptoms [67]. Therefore, two psychological indicators, anxiety and depression, were used to judge the effect of self-management education on patients’ mental health in this study. However, the overall analysis found that chronic disease self-management interventions did not significantly alleviate patients’ anxiety symptoms, which is contrary to the findings of a previous study [68]. A study found that self-management interventions have a significant positive impact on anxiety symptoms in patients with chronic kidney disease. The explanation for such results may be that patients’ anxiety symptoms are susceptible to external factors, and the anxiety measurement tool is not sensitive enough to exclude the influence of other factors. After subgroup analysis, it was found that self-management interventions with follow-up times of less than 6 months were effective in improving patients’ anxiety symptoms. During self-management interventions, the more patients learn, the more they worry that they will not be able to master disease self-management, so the longer it takes the more anxious they become. Because there are no standardized criteria for self-management interventions for people with chronic diseases, interventions and follow-up times vary widely, leading to mixed results. Thus, there is also a requirement to clarify the particulars of self-management interventions for the reduction of patient anxiety in the future. Additionally, the results of this study showed that self-management interventions reduced depressive symptoms in chronic disease patients, in line with the findings of a meta-analysis [69]. Research has reported that self-management intervention includes emotional management, mental health education, and other courses, which can promote the reduction of depressive symptoms [21]. Moreover, improving physical activity and adopting a healthy and conscious diet may reduce symptoms of depression [69]. As the health care costs, disease progression, and comorbidities of chronic diseases will affect the mental health of patients, it is necessary to develop a complete and personalized emotional self-management program.

There are many types of self-management interventions, including hybrid forms of interventions. The studies included in this paper do not encompass all intervention types and their corresponding data. Additionally, even within the same type of intervention, variations in dosage (such as the duration of each session) and frequency (such as the number of sessions per week) can result in different outcomes. This paper does not determine which intervention type or hybrid intervention type is most effective. We see great promise in the type of intervention based on Internet and mHealth technologies self-management. The knowledge and skills that are acquired through the mobile health application are important and necessary for improving disease self-management behavior in patients [37]. In the future, a network platform that is more conducive to patients’ self-management can be developed. In addition, other types of self-management interventions can be combined to maximize patient support for disease self-management.

Chronic disease patients often face ongoing health challenges. These conditions are characterized by a long duration, complex etiology, diverse symptoms, and a tendency to recur. These characteristics not only lead to adverse health outcomes, but also significantly impact the patients’ quality of life and increase healthcare costs. Therefore, healthcare providers need to develop effective management plans for chronic disease patients to help them better manage their conditions and improve their quality of life. This study demonstrates that self-management interventions for chronic diseases are more effective for routine care. These interventions can help patients adapt to the changes brought about by the disease and self-manage diseases to prevent disease progression. At the same time, self-management interventions help patients improve their mental health and reduce the utilization of healthcare resources by supporting patients in actively adjusting their health behaviors and developing better skills to manage their diseases, thereby improving patient outcomes and reducing the disease burden.

Despite the important findings of this study, there are several limitations that need to be considered. Firstly, the 34 included studies varied significantly in terms of intervention content and methods, assessment tools, and risk of bias control. Not all chronic disease categories were reflected in the samples of these included studies. The geographical distribution was limited, with most studies coming from the USA, UK, and China, which may affect the generalizability of the intervention effects. Among various interventions, researchers may be more inclined to report positive outcomes based on new technologies, while overlooking the effects of traditional interventions. This may have led to publication bias in quality of life outcomes, potentially overestimating the effectiveness of self-management interventions and increasing heterogeneity. Secondly, while this study was unable to determine which form of intervention is more effective in improving specific outcomes, future research could employ network meta-analysis or include additional studies to clarify the relative effectiveness of different treatment approaches. While this study did not identify which form of self-management intervention is most effective, combining multiple intervention methods (such as health coaching, group meetings, and internet or mobile health technologies) can enhance patient engagement and adherence [12]. Additionally, the mechanism of the effect of self-management interventions on the mental health of patients with chronic diseases has not been fully clarified. Future research should further investigate the impact of self-management interventions on anxiety. While this study found significant improvements in depression, similar significant differences were not observed for anxiety. This may require larger sample sizes, more diverse patient populations, and different intervention methods to better understand the mechanisms and effects of these interventions in reducing anxiety. Therefore, future studies should clarify its effects on mental health and the interplay between them. As mentioned in the previous article, healthy lifestyle choice strategies are beneficial to patients, but most of the existing studies are single-disease strategies, and future studies could be designed in the direction of multiple diseases. It is also important to consider the types of self-management interventions and what interventions are most effective. According to the results of this study, patients with chronic diseases who are older than 60 years of age have overall better outcome indicators. Later, more attention should also be paid to how to motivate the patients with chronic diseases of other age groups to engage in disease self-management for a better disease prognosis. Follow-up times longer than 6 months increase patients’ quality of life, but does not improve their anxiety symptoms. In the case of this study, it does present the benefits of a follow-up time greater than 6 months for patients, but fails to explain whether patients’ anxiety symptoms are really closely related to the follow-up time. Future studies should also focus on the effect of follow-up time on patients in order to set an optimal follow-up time. In a word, we should think about how to improve the effectiveness of the self-management intervention to improve the quality of life and self-efficacy of patients and reduce patients’ anxiety and depression. Consequently, future research can explore and optimize these comprehensive intervention strategies to improve patients’ self-management capabilities and overall outcomes. At the same time, it is hoped that a tracking system can be set up in the future to follow-up and remind patients when they are prone to slackness in disease self-management, and to give them appropriate help.

## 5. Conclusions

This study found that self-management interventions have positive effects on patients’ quality of life, self-efficacy, and mental health, providing strong support for the care and management of chronic disease patients. For example, various forms of self-management interventions can be personalized based on the specific needs of patients. Subgroup analysis revealed that patients with an average age over 60 years benefit significantly from long-term follow-up, particularly in terms of quality of life (Lower-better QOL) and mental health (depression and anxiety). Consequently, healthcare providers should consider increasing long-term follow-up support and intervention measures for elderly chronic disease patients to ensure sustained treatment effects.

## Data Availability

The original contributions presented in the study are included in the article/Supplementary Materials, further inquiries can be directed to the corresponding author/s.

## Supplementary Materials

The following supporting information can be downloaded at: https://www.mdpi.com/article/10.3390/healthcare12212151/s1. Table S1: Basic information of included studies; Table S2: Characteristics of the self-management intervention in each study; Table S3: Results of subgroup analyses of Higher-better QOL, Lower-better QOL, SE, Depression and Anxiety; Figure S1: Forest plot: Subgroup analysis were performed based on mean age to explore its effect on Low-better QOL; Figure S2: Forest plot: Subgroup analysis were performed based on follow-up times to explore its effect on self-efficacy; Supplementary Material S1: Detailed search strategy; Supplementary Material S2: PRISMA Checklist.
