# Supplementary material for: The Effect of Self-Management on Patients with Chronic Diseases: A Systematic Review and Meta-Analysis
Source: Healthcare (Basel). 2024 Oct 29;12(21):2151. doi: 10.3390/healthcare12212151 (PMC11544912; doi:10.3390/healthcare12212151)
Supplement: Supplementary file 1 [file healthcare-12-02151-s001.zip › Search Strategy.pdf]

Index Terms and Keywords for searching in three databases

<u>Database: PubMed</u> (1922 records found)

(Chronic disease OR chronic illness OR NCD OR non communicable disease OR Heart disease OR heart failure OR cardiac disease OR cardiac failure OR cardiovascular disease OR cardiovascular disorder OR cardio-vascular disease OR cardio-vascular disorder OR CVD OR chronic heart failure OR congestive heart failure OR CHF OR coronary heart disease OR CHD OR angina OR myocardial infarction OR Hypertension OR high blood pressure OR HTN OR TIA OR stroke OR cerebral hemorrhage OR hematencephalon OR cerebral infarction OR Parkinson’s disease OR PD OR Parkinsonism OR obesity OR overweight OR overnutrition OR High cholesterol OR hypercholesterolemia OR dyslipidemia OR diabetes OR pre-diabetes OR DM OR T2DM OR T1DM ORNIDD OR IDD OR hyperglycemia OR High glucose OR chronic obstructive pulmonary disease OR chronic respiratory diseases OR CRD OR COPD OR pulmonary disease OR pulmonary illness OR pulmonary failure OR lung disease OR lung illness OR lung failure OR chronic asthma OR bronchiectasia OR chronic bronchitis OR Intestinal disease OR intestinal disorder OR intestinal illness OR digestive disease OR digestive disorder OR digestive illness OR chronic gastroenteritis OR ulcerative disease OR ulcerative illness OR ulcerative colitis OR UC OR gastric ulcer OR crohn disease OR GERD OR fatty liver OR chronic renal diseases OR chronic kidney disease OR CKD OR renal insufficiency OR Kidney insufficiency OR chronic renal impairement OR chronic kidney impairement OR chronic renal failure OR CRF OR chronic kidney failure OR end stage kidney disease OR ESKD OR end stage renal disease OR ESRD OR end stage kidney failure OR ESKF OR end stage renal failure OR ESRF OR chronic cystitis OR rheumatoid arthritis) AND self-management

Filter: Publication Year：2016-2021; Article type: controlled clinical trial and randomized controlled trial

<u>Database: Embase </u>(1228 records found)

('chronic disease'/exp OR 'chronic disease' OR 'chronic illness' OR 'heart disease'/exp OR 'cardiac anomaly' OR 'cardiac disease' OR 'cardiac disturbance' OR 'cardiopathy' OR 'heart deficiency' OR 'heart deformity' OR 'heart disease' OR 'heart diseases' OR 'heart disorder' OR 'heart dysfunction' OR 'hypertension'/exp OR 'htn (hypertension)' OR 'acute hypertension' OR 'arterial hypertension' OR 'blood pressure, high' OR 'cardiovascular hypertension' OR 'controlled hypertension' OR 'endocrine

hypertension' OR 'high blood pressure' OR 'high renin hypertension' OR 'hypertension' OR 'hypertensive disease' OR 'hypertensive effect' OR 'hypertensive response' OR 'neurogenic hypertension' OR 'preexistent hypertension' OR 'salt high blood pressure' OR 'salt hypertension' OR 'secondary hypertension' OR 'systemic hypertension' OR 'cerebrovascular accident'/exp OR 'cva' OR 'accident, cerebrovascular' OR 'apoplectic stroke' OR 'apoplexia' OR 'apoplexy' OR 'brain accident' OR 'brain blood flow disturbance' OR 'brain vascular accident' OR 'cerebral apoplexia' OR 'cerebral stroke' OR 'cerebral vascular accident' OR 'cerebral vascular insufficiency' OR 'cerebro vascular accident' OR 'cerebrovascular accident' OR 'cerebrovascular failure' OR 'cerebrovascular insufficiency' OR 'cerebrum vascular accident' OR 'cryptogenic stroke' OR 'ischaemic seizure' OR 'ischemic seizure' OR 'stroke' OR 'thrombotic stroke' OR 'brain infarction'/exp OR 'brain cortex infarct' OR 'brain cortex infarction' OR 'brain infarct' OR 'brain infarction' OR 'cerebral cortex infarct' OR 'cerebral cortex infarction' OR 'cerebral infarct' OR 'cerebral infarction' OR 'cerebrovascular infarct' OR 'cerebrovascular infarction' OR 'cortical infarct' OR 'cortical infarction' OR 'hemisphere infarct' OR 'hemisphere infarction' OR 'hemispheric infarct' OR 'hemispheric infarction' OR 'infarction, brain' OR 'silent brain infarction' OR 'brain hemorrhage'/exp OR 'obesity'/exp OR 'adipositas' OR 'adiposity' OR 'alimentary obesity' OR 'corpulency' OR 'fat overload syndrome' OR 'nutritional obesity' OR 'obesitas' OR 'obesity' OR 'overweight' OR 'diabetes mellitus'/exp OR 'diabetes' OR 'diabetes mellitus' OR 'diabetic' OR 'chronic lung disease'/exp OR 'chronic lung disease' OR 'chronic pneumopathy' OR 'chronic pulmonary disease' OR 'lung chronic disease' OR 'lung disease, chronic' OR 'bronchiolitis'/exp OR 'bronchiolitis' OR 'capillary bronchiolitis' OR 'chronic obstructive lung disease'/exp OR 'chronic airflow obstruction' OR 'chronic airway obstruction' OR 'chronic obstructive bronchopulmonary disease' OR 'chronic obstructive lung disease' OR 'chronic obstructive lung disorder' OR 'chronic obstructive pulmonary disease' OR 'chronic obstructive pulmonary disorder' OR 'chronic obstructive respiratory disease' OR 'chronic pulmonary obstructive disease' OR 'chronic pulmonary obstructive disorder' OR 'copd' OR 'lung chronic obstructive disease' OR 'lung disease, chronic obstructive' OR 'obstructive chronic lung disease' OR 'obstructive chronic pulmonary disease' OR 'obstructive lung disease, chronic' OR 'pulmonary disease, chronic obstructive' OR 'pulmonary disorder, chronic obstructive' OR 'bronchiectasis'/exp OR 'bronchiectasia' OR 'bronchiectasis' OR 'bronchoectasia' OR 'congenital bronchiectasis' OR 'asthma'/exp OR 'asthma' OR 'asthma bronchiale' OR 'asthma pulmonale' OR 'asthma, bronchial' OR 'asthmatic' OR 'asthmatic subject' OR 'bronchial asthma' OR 'bronchus asthma' OR 'chronic asthma' OR 'lung allergy' OR 'enteropathy'/exp OR 'bowel disease' OR 'bowel disorder' OR 'disorder, intestine' OR 'enteropathy' OR 'gut disease' OR 'intestinal disease' OR 'intestinal diseases' OR 'intestinal disorder' OR 'intestinal tract disease' OR 'intestine disease' OR 'intestine disorder' OR 'chronic gastritis'/exp OR 'chronic cystic gastritis' OR 'chronic gastritis' OR 'gastritis, chronic' OR 'ulcerative disease' OR 'ulcerative colitis'/exp OR 'chronic ulcerative colitis' OR 'colitis ulcerativa' OR 'colitis ulcerosa' OR 'colitis ulcerosa chronica' OR 'colitis, mucosal' OR 'colitis, ulcerative' OR 'colitis, ulcerous' OR 'colon,

chronic ulceration' OR 'histiocytic ulcerative colitis' OR 'mucosal colitis' OR 'ulcerative colitis' OR 'ulcerative colorectitis' OR 'ulcerative procto colitis' OR 'ulcerative proctocolitis' OR 'ulcerous colitis' OR 'stomach ulcer'/exp OR 'acute gastric ulcer' OR 'gastric peptic ulcer' OR 'gastric ulcer' OR 'gastric ulceration' OR 'gastric ulcus' OR 'peptic stomach ulcer' OR 'peptic ulcer, gastric' OR 'peptic ulcer, stomach' OR 'stomach bleeding ulcer' OR 'stomach peptic ulcer' OR 'stomach ulcer' OR 'stomach ulceration' OR 'stomach ulcus' OR 'stomach ulcus callosum' OR 'ulcer, stomach' OR 'ulcus callosum' OR 'ulcus ventriculi' OR 'ulcus, stomach' OR 'ventricular ulcer' OR 'crohn disease'/exp OR 'fatty liver'/exp OR 'fat liver' OR 'fatty change in liver' OR 'fatty infiltration of liver' OR 'fatty liver' OR 'fatty liver disease' OR 'fatty liver infiltration' OR 'fatty liver syndrome' OR 'fatty metamorphosis of the liver' OR 'hepatic lipidosis' OR 'hepatic steatosis' OR 'hepato-steatosis' OR 'hepatolipidosis' OR 'hepatosteatosis' OR 'liver fatty change' OR 'liver fatty infiltration' OR 'liver lipidosis' OR 'liver steatosis' OR 'liver, fatty' OR 'steato-hepatosis' OR 'steatohepatopathy' OR 'steatohepatosis' OR 'steatosis, liver' OR 'steatotic hepatopathy' OR 'steatotic liver' OR 'chronic kidney failure'/exp OR 'chronic kidney disease' OR 'chronic kidney disorder' OR 'chronic kidney failure' OR 'chronic kidney insufficiency' OR 'chronic nephropathy' OR 'chronic renal disease' OR 'chronic renal failure' OR 'chronic renal insufficiency' OR 'kidney chronic failure' OR 'kidney disease, chronic' OR 'kidney failure, chronic' OR 'kidney function, chronic disease' OR 'renal insufficiency, chronic' OR 'chronic cystitis'/exp OR 'rheumatoid arthritis'/exp OR 'arthritis deformans' OR 'arthritis, rheumatoid' OR 'arthrosis deformans' OR 'beauvais disease' OR 'chronic articular rheumatism' OR 'chronic polyarthritis' OR 'chronic progressive poly arthritis' OR 'chronic progressive polyarthritis' OR 'chronic rheumatoid arthritis' OR 'disease, beauvais' OR 'infantile rheumatoid arthritis' OR 'inflammatory arthritis' OR 'polyarthritis, primary chronic' OR 'primary chronic polyarthritis' OR 'rheumarthritis' OR 'rheumatic arthritis' OR 'rheumatic polyarthritis' OR 'rheumatism, chronic articular' OR 'rheumatoid arthritis') AND ('self care'/exp OR 'self care' OR 'self management' OR 'self treatment' OR 'self-management' OR 'self-nurturance' OR 'selfcare' OR 'selfmanagement' OR 'selftreatment') AND ('quality of life'/exp OR 'hrql' OR 'health related quality of life' OR 'life quality' OR 'quality of life' OR 'self efficacy' OR 'anxiety'/exp OR 'anxiety' OR 'depression'/exp OR 'depression' OR 'depressive state' OR 'depressive symptom') AND 'randomized controlled trial'/exp AND [2016-2021]/py

Database: Web of science (1238 records found)

(Chronic disease OR chronic illness OR NCD OR non communicable disease OR Heart disease OR heart failure OR cardiac disease OR cardiac failure OR cardiovascular disease OR cardiovascular disorder OR cardio-vascular disease OR cardio-vascular disorder OR CVD OR chronic heart failure OR congestive heart failure OR CHF OR coronary heart disease OR CHD OR angina OR myocardial infarction OR Hypertension OR high blood pressure OR HTN OR TIA OR stroke OR cerebral hemorrhage OR hematencephalon OR cerebral infarction OR Parkinson’s

disease OR PD OR Parkinsonism OR obesity OR overweight OR overnutrition OR High cholesterol OR hypercholesterolemia OR dyslipidemia OR diabetes OR pre-diabetes OR DM OR T2DM OR T1DM ORNIDD OR IDD OR hyperglycemia OR High glucose OR chronic obstructive pulmonary disease OR chronic respiratory diseases OR CRD OR COPD OR pulmonary disease OR pulmonary illness OR pulmonary failure OR lung disease OR lung illness OR lung failure OR chronic asthma OR bronchiectasia OR chronic bronchitis OR Intestinal disease OR intestinal disorder OR intestinal illness OR digestive disease OR digestive disorder OR digestive illness OR chronic gastroenteritis OR ulcerative disease OR ulcerative illness OR ulcerative colitis OR UC OR gastric ulcer OR crohn disease OR GERD OR fatty liver OR chronic renal diseases OR chronic kidney disease OR CKD OR renal insufficiency OR Kidney insufficiency OR chronic renal impairement OR chronic kidney impairement OR chronic renal failure OR CRF OR chronic kidney failure OR end stage kidney disease OR ESKD OR end stage renal disease OR ESRD OR end stage kidney failure OR ESKF OR end stage renal failure OR ESRF OR chronic cystitis OR rheumatoid arthritis) AND self-management

Filter: Publication Year：2016-2021; Article type: clinical trial
