# Supplementary material for: The Effect of Self-Management on Patients with Chronic Diseases: A Systematic Review and Meta-Analysis
Source: Healthcare (Basel). 2024 Oct 29;12(21):2151. doi: 10.3390/healthcare12212151 (PMC11544912; doi:10.3390/healthcare12212151)
Supplement: Supplementary file 1 [file healthcare-12-02151-s001.zip › Supplementary Materials.pdf]

Table S1. Basic information of included studies.

Author	Year	Country	Type of chronic diseases	Sample size (data analysis size)	Mean Age	Men(%)	Follow-up time	Primary outcomes	Forms of self-management interventions	Definition of intervention	Definition of control
Jolly et al. (Jolly et al., 2018)	2018	UK	COPD	577（528）	70.4	63.4	12 months	Quality of life; self-efficacy; anxiety; depression	Telephone health coaching	Supporting written documents；a pedometer；self monitoring diary	A standard information leaflet about self-management of COPD
Aboumatar et al. (Aboumatar et al., 2019)	2019	USA	COPD	240（239）	65	38.3	6 months	Quality of life	Group meeting; Health coaching	Transition support; individualized COPD self-management support; facilitated access to community programs and treatment services	The usual transitional care
Salisbury et al. (Salisbury et al., 2018)	2018	UK	Multimorbidity	1546	70.8	49.4	15 months	Quality of life; anxiety; depression	Based on Internet and mhealth technologies; Health coaching	Nurse review; pharmacist review; physician review	The usual care
Towfighi	2021	USA	Stroke	487	57.1	65.1	12	Quality of life;	Based on	Hypothesized core	Free BP monitors;

et al. (Towfighi, Cheng, Ayala-Rivera, Barry, McCreath, Ganz, Lee, Sanossian, Mehta, Dutta, Razmara, Bryg, & Song, 2021)			or TIA				months	self-efficacy; depression	Internet and mhealth technologies; Health coaching	components; telephone visits; risk factor management; education materials; self-management tools	self-management tools (BP and glucose logs); educational materials
Rahimi et al. (Rahimi et al., 2020)	2020	UK	CHF	202 (173)	71.6	71.8	6 months	Quality of life	Based on Internet and mhealth technologies	Digital home monitoring with centralised specialist support for remote management of HF	Digital home monitoring
Dang et al. (Dang, Karanam, & Gomez-Marin,	2017	USA	CHF	61 (52)	55.3	63.9	3 months	Quality of life; self-efficacy	Based on Internet and mhealth technologies; Telephone health	Daily questions sent through Web Browser Messaging	The usual care

2017)									coaching		
Wu et al. (Wu et al., 2018)	2018	China	Pre-end-stage renal disease	112	70.16	61.6	3 months	Self-efficacy; anxiety; depression	Group meeting	The course (a video; a group trainee manual about self-efficacy; the management of CKD; the self-efficacy-enhancing program; telephone interview)	Routine blood tests every 3 months; traditional health education;
Markle-Reid et al. (Markle-Reid et al., 2018)	2018	Canada	T2DM	159	-	44.0	6 months	Quality of life; self-efficacy; anxiety; depression	Family visit; Group meeting	Offer up to 3 in-home visits by the RN, RD; monthly group wellness sessions; monthly case conferences	The usual care
Miklavcic et al. (Miklavcic et al., 2020)	2020	Canada	T2DM and multimorbidity	132	-	45	6 months	Quality of life; self-efficacy; anxiety; depression	Family visit; Group meeting	Training/educational workshops; monthly implementation meetings; reminders; audit and feedback	The usual care
Still et al. (Still et al., 2020)	2020	USA	Hypertension	60	60	25	3 months	Self-efficacy	Based on Internet and mhealth technologies; Telephone health coaching	Web-based hypertension education; self-monitoring BP; medication management support; 30 minutes nurse	Printed educational materials; one web-based education session

										counseling sessions	
Wang1 et al. (L. Wang, Guo, Wang, & Zhao, 2021)	2021	China	COPD	78	63.9	70.5	12 months	Quality of life; self-efficacy	Based on Internet and mhealth technologies	A mobile health application program	Health education
Wang2 et al. (L. H. Wang, Zhao, Chen, Zhang, & Zhang, 2020)	2020	China	COPD	154	69.0	78.6	12 months	Quality of life	Group meeting; Family visit	Access general COPD and physical activity self-management needs; five to six face-to-face, individually tailored education sessions; phone calls every week and three home visits	Health education for COPD
Newhouse et al. (Newhouse et al., 2016)	2016	UK	Chronic asthma	148	57.0	41.2	2 weeks	Self-efficacy	Based on Internet and mhealth technologies	Browse examples of the 25 topics in a multimedia website	The usual care
Zhang et al. (Zhang et al., 2020)	2020	China	COPD	208 (174)	65.93	75.9	24 months	Quality of life	Group meeting; Telephone health	Exercise; smoking cessation; self-management training; psychosocial	The usual care; a Pamphlet about COPD

									coaching or family visit	support; telephone follow-up and home visits	
Griva et al. (Griva et al., 2018)	2018	Singapor e	Hemod ialysis	235	53.5	58.3	9 months	Quality of life; self-efficacy	Group meeting; Telephone health coaching	Over 3 core sessions plus 1 booster session; an additional telephone follow-up call; educational booklet	Standard renal care; educational booklet
Nguyen et al. (Nguyen, Douglas, & Bonner, 2019)	2019	Vietnam	CKD	135	48.9	50.3	16 weeks	Quality of life; self-efficacy	Group meeting; Telephone health coaching	A CKD booklet; handout; one face-to-face session; two brief follow-up sessions	2-5 minutes brief verbal information
Jiang et al. (Jiang et al., 2020)	2020	China	COPD	106	71.4	82.1	6 months	Quality of life	Based on Internet and mhealth technologies	Pulmonary rehabilitation intervention program; doctor-patient interaction through a WeChat official account(PeR)	The same intervention as the PeR group Without using WeChat
Park et al. (Park, Bang, &	2020	Korea	COPD	42	67.88	78.6	6 months	Quality of life; self-efficacy; anxiety; depression	Based on Internet and mhealth technologies	A smartphone app-based self-management program (education, exercises,	One call a month from the research staff

Lee, 2020)										self-monitoring of symptoms and exercise, and social support)	
Pauley et al. (Pauley, Gargaro, Chenard, Cavanagh, & McKay, 2016)	2016	Canada	Diabetes	94	66	51.5	6 weeks	Anxiety; depression	Health coaching	Six 1-hour one-to-one in-home coaching sessions	Standard diabetes care
Dorsch et al. (Dorsch, Farris, Rowell, Hummel, & Koelling, 2021)	2021	USA	CHF	83	61.1	65.1	12 weeks	Quality of life	Based on Internet and mhealth technologies	Use a mobile app (ManageHF4Life), a fitbit physical activity monitor and scale to daily self-monitoring	The usual care
Farmer et al. (Farmer et al., 2017)	2017	UK	COPD	166 (141)	69.8	61.4	12 months	Quality of life	Based on Internet and mhealth technologies	Write the symptom diary and use pulse oximeter with finger probe to self monitor	provide with all the information given to those allocated to use the

										through EDGE	EDGE system
Boer et al. (Boer et al., 2019)	2019	Netherlands	COPD	87 (76)	67.6	62.1	12 months	Quality of life; self-efficacy	Based on Internet and mhealth technologies	Use mobile phone to answer questions and the mHealth tool provide related advice	A paper exacerbation action plan
Kalav et al. (Kalav, Bektas, & Ünal, 2021)	2021	Turkey	Ischemic stroke	68	57.4	67.4	12 weeks	Quality of life; self-efficacy	Health coaching	The education booklet and blood pressure follow-up form; telephone follow-ups and sending reminder messages	The routine patient care
Brockwell et al. (Brockwell et al., 2020)	2020	UK	Bronchiectasis	220	66.9	31	12 months	Quality of life	Based on Internet and mhealth technologies; Telephone health coaching	The BET document (an action plan, four educational support sections); education sessions	The usual care
Walker et al. (Walker et al., 2018)	2018	Multicenter	COPD	312	-	66	9 months	Quality of life; depression	Based on Internet and mhealth technologies	Used the CHROMED monitoring platform (self-assessed lung mechanics daily; monitor cardiac parameters; An algorithm identified	The usual care

										deterioration, triggering a telephone contact to determine appropriate interventions)	
Dalal et al. (Dalal et al., 2019)	2019	Multicenter	HF	216（185）	70	78	12 months	Quality of life; anxiety; depression	Printed materials; Health coaching	Medical management and care of heart failure; REACH-HF intervention	Medical management and care of heart failure
Wang et al. (W. Wang et al., 2018)	2018	Singapore	CHD	129	60.8	89.1	16 weeks	Anxiety; depression	Printed materials; Health coaching	A newly developed booklet, a digital video disc, an individual face-to-face education session and four telephone follow-ups	The usual care
Young et al. (Young, Miyamoto, Dharmar, & Tang-Feldman, 2020)	2020	USA	T2DM	319	59.1	52.7	9 months	Self-efficacy; anxiety; depression	Health coaching; Based on Internet and mhealth technologies	Nurse health coaching and mHealth technology to track patient-generated health data	The usual care

Rieger et al. (Rieger, Treasure, Murray, & Caterson, 2017)	2017	Australia	obesity	201（200）	47.0	26.4	12 months	Quality of life; self-efficacy	Group meeting	One-year cognitive behavior therapy (CBT) weight management program with the addition of support people	One-year cognitive behavior therapy (CBT) weight management program alone
Pack et al. (Pack & Lee, 2021)	2021	Korea	haemo dialysis	75	51.3	61.3	12 weeks	Quality of life; self-efficacy	Health coaching; Based on Internet and mhealth technologies	Face-to-face training; online counseling; aid dietary management through the smartphone application-based dietary self-management program	Face-to-face training ;general dietary program; online counseling
Gong et al. (Gong et al., 2020)	2020	Australia	T2DM	187（145）	57.0	58.3	12 months	anxiety; depression	Based on Internet and mhealth technologies; Telephone health coaching	The MDC app; a printed user guide; the MDC website; an optional blood glucose meter with Bluetooth capability; interactions with a program coordinator	Routine diabetes self-care
Ahmed et al.	2016	Canada	Asthma	98（75）	-	33.7	9months	Quality of life	Based on Internet and	Acquire core knowledge and skills	The usual care

(Ahmed et al., 2016)									mhealth technologies	through MAP	
Zeng et al. (Zeng, Yang, & Chien, 2021)	2021	China	Hypertension	44	63.1	45.5	10 weeks	Quality of life; self-efficacy; anxiety; depression	Health coaching	An education workshop of hypertension and its management; a series of dyadic partnership skills training sessions	Receive usual care delivered by a village doctor
Leenen et al. (Leenen et al., 2018)	2018	Netherlands	epilepsy	102（96）	41.7	49	6 months	Quality of life; self-efficacy; anxiety; depression	Group meeting; Based on Internet and mhealth technologies	Five weekly group sessions; two-hour booster session after 3 weeks	The usual care

Table S2. Characteristics of the self-management intervention in each study

Author	Diet and nutrition	Physical activity	Medication management	Emotional regulation	Disease-related knowledge	Smoking cessation	Action Plan	Disease self-monitoring	Assessment of disease progression
Jolly et al. (Jolly et al., 2018)		√	√		√	√	√	√	
Aboumatar et al. (Aboumatar et al., 2019)		√	√			√	√		√
Salisbury et al. (Salisbury et al., 2018)			√	√	√	√	√		
Towfighi et al. (Towfighi, Cheng, Ayala-Rivera,			√		√		√	√	

Barry, McCreath, Ganz, Lee, Sanossian, Mehta, Dutta, Razmara, Bryg, Song, et al., 2021)								
Rahimi et al. (Rahimi et al., 2020)			√		√		√	
Dang et al. (Dang et al., 2017)	√		√				√	√
Wu et al. (Wu et al., 2018)				√	√	√		
Markle-Reid et al. (Markle-Reid et al.,	√	√		√	√	√		

2018)									
Miklavcic et al. (Miklavcic et al., 2020)	√	√			√		√		
Still et al. (Still et al., 2020)	√	√	√		√			√	
Wang1 et al. (L. Wang et al., 2021)		√	√	√	√				
Wang2 et al. (L. H. Wang et al., 2020)		√	√	√	√	√	√		
Newhouse et al. (Newhouse et al., 2016)				√	√	√			
Zhang		√	√	√	√	√		√	√

et al. (Zhang et al., 2020)							
Griva et al. (Griva et al., 2018)	√		√		√	√	
Nguyen et al. (Nguyen et al., 2019)	√	√		√	√	√	
Jiang et al. (Jiang et al., 2020)	√	√	√				√
Park et al. (Park et al., 2020)		√		√	√		√
Pauley et al. (Pauley et al., 2016)			√	√	√	√	√
Dorsch et al.		√			√		√

(Dorsch et al., 2021)							
Farmer et al. (Farmer et al., 2017)		√	√	√		√	
Boer et al. (Boer et al., 2019)		√	√	√		√	
Kalav et al. (Kalav et al., 2021)				√	√	√	
Brockwell et al. (Brockwell et al., 2020)		√		√		√	√
Walker et al. (Walker et al., 2018)						√	
Dalal et al.	√	√	√	√		√	

(Dalal et al., 2019)								
Wang et al. (W. Wang et al., 2018)	√	√			√			√
Young et al. (Young et al., 2020)	√	√	√			√		
Rieger et al. (Rieger et al., 2017)	√	√		√		√	√	
Pack et al. (Pack & Lee, 2021)	√							
Gong et al. (Gong et al., 2020)	√	√	√	√			√	

Ahmed et al. (Ahmed et al., 2016)			√		√		√		
Zeng et al. (Zeng et al., 2021)	√	√	√	√	√	√	√		
Leenen et al. (Leenen et al., 2018)			√				√	√	√

Table S3. Results of subgroup analyses of Higher-better QOL, Lower-better QOL, SE, Depression and Anxiety

Variable	Studies, n	SMD	95%CI	I^2(%)	*P* value
Higher-better QOL					
Mean age					
<60 years old	3	0.23	[-0.12,0.59]	46	0.20
≥60 years old	5	0.08	[-0.00,0.17]	0	0.06
Follow-up times					
≤6 months	3	0.12	[-0.30,0.53]	50	0.59
>6 months	7	0.07	[-0.01,0.14]	0	0.07
Forms of self-management					
Health coaching	3	0.03	[-0.13,0.19]	0	0.69
Group meeting	0	N/A	N/A	N/A	N/A
Based on Internet and mhealth technologies	3	0.06	[-.013,0.24]	0	0.54
Group meeting & Health coaching	0	N/A	N/A	N/A	N/A
Group meeting & Family visit	0	N/A	N/A	N/A	N/A
Based on Internet and mhealth technologies & Health coaching	3	0.10	[-0.06,0.26]	50	0.22
Printed materials & Health coaching	1	0.05	[-0.24,0.34]	N/A	0.73
Group meeting & Health	0	N/A	N/A	N/A	N/A

coaching/ Family visit					
Group meeting & Based on Internet and mhealth technologies	1	0.32	[-0.10,0.75]	N/A	0.14
Lower-better QOL					
Mean age					
<60 years old	2	-0.25	[-0.50,0.01]	0	0.06
≥60 years old	9	-0.32	[-.061,-0.03]	85	0.03
Follow-up times					
≤6 months	4	-0.08	[-0.37,0.21]	54	0.61
>6 months	6	-0.48	[-0.80,-0.16]	83	0.004
Forms of self-management					
Health coaching	0	N/A	N/A	N/A	N/A
Group meeting	1	-0.28	[-0.56,-0.01]	N/A	0.05
Based on Internet and mhealth technologies	5	-0.20	[-0.44,0.04]	50	0.10
Group meeting & Health coaching	1	0.19	[-0.06,0.45]	N/A	0.13
Group meeting & Family visit	1	-0.87	[-1.20,-0.54]	N/A	<0.001
Based on Internet and mhealth technologies & Health coaching	1	-0.08	[-0.66,0.51]	N/A	0.80
Printed materials & Health coaching	1	-0.15	[-0.44,0.14]	N/A	0.30
Group meeting & Health coaching/ Family visit	1	-0.96	[-1.28,-0.65]	N/A	<0.001

Group meeting & Based on Internet and mhealth technologies	0	N/A	N/A	N/A	N/A
		Self-efficacy			
Mean age					
<60 years old	10	0.22	[0.01,0.43]	77	0.04
≥60 years old	7	0.76	[0.16,1.36]	93	0.01
Follow-up times					
≤6 months	12	0.46	[0.10,0.82]	88	0.01
>6 months	7	0.31	[0.05,0.56]	86	0.02
Forms of self-management					
Health coaching	3	0.21	[-0.29,0.70]	77	0.41
Group meeting	2	1.18	[-0.77,3.13]	98	0.23
Based on Internet and mhealth technologies	4	0.47	[-.031,1.25]	91	0.24
Group meeting & Health coaching	2	0.63	[-0.06,1.32]	90	0.07
Group meeting & Family visit	2	0.19	[-0.05,0.42]	0	0.13
Based on Internet and mhealth technologies & Health coaching	4	0.05	[-0.13,0.22]	29	0.59
Printed materials & Health coaching	0	N/A	N/A	N/A	N/A
Group meeting & Health coaching/ Family visit	0	N/A	N/A	N/A	N/A
Group meeting & Based on	1	0.34	[-0.09,0.76]	N/A	0.12

Internet and mhealth technologies					
		Depression			
Mean age					
<60 years old	4	-0.20	[-0.37,-0.03]	39	0.02
≥60 years old	9	-0.16	[-0.28,-0.04]	32	0.009
Follow-up times					
≤6 months	9	-0.19	[-0.36,-0.02]	31	0.03
>6 months	7	-0.14	[-0.23,-0.05]	32	0.002
Forms of self-management					
Health coaching	3	-0.13	[-0.29,0.02]	0	0.09
Group meeting	1	-0.66	[-1.05,-0.28]	N/A	<0.001
Based on Internet and mhealth technologies	2	-0.02	[-0.34,0.29]	28	0.88
Group meeting & Health coaching	0	N/A	N/A	N/A	N/A
Group meeting & Family visit	2	-0.20	[-0.44,0.04]	0	0.10
Based on Internet and mhealth technologies & Health coaching	5	-0.16	[-0.30,-0.02]	50	0.02
Printed materials & Health coaching	2	-0.10	[-0.33,0.12	0	0.37
Group meeting & Health coaching/ Family visit	0	N/A	N/A	N/A	N/A
Group meeting & Based on Internet and mhealth	1	-0.11	[-0.53,0.31]	N/A	0.61

technologies					
		Anxiety			
Mean age					
<60 years old	2	-0.05	[-0.46,0.36]	77	0.82
≥60 years old	8	-0.07	[-0.20,0.07]	42	0.33
Follow-up times					
≤6 months	7	-0.17	[-0.32,-0.02]	0	0.03
>6 months	5	-0.02	[-0.18,0.14]	68	0.84
Forms of self-management					
Health coaching	3	0.11	[-0.05,0.27]	37	0.17
Group meeting	1	-0.40	[-0.78,-0.02]	N/A	0.04
Based on Internet and mhealth technologies	1	-0.14	[-0.75,0.46]	N/A	0.65
Group meeting & Health coaching	0	N/A	N/A	N/A	N/A
Group meeting & Family visit	2	-0.18	[-0.41,0.05]	0	0.13
Based on Internet and mhealth technologies & Health coaching	3	-0.08	[-0.17,0.01]	65	0.08
Printed materials & Health coaching	2	-0.06	[-0.29,0.16]	0	0.58
Group meeting & Health coaching/ Family visit	0	N/A	N/A	N/A	N/A
Group meeting & Based on Internet and mhealth technologies	0	N/A	N/A	N/A	N/A

Study or Subgroup	Experimental Mean	Experimental SD	Experimental Total	Control Mean	Control SD	Control Total	Weight	Std. Mean Difference IV, Random, 95% CI
**5.6.1 ≤6 months**								
Dang et al.	7.27	1.91	36	6.08	1.9	16	4.3%	0.61 [0.01, 1.22]
Kalav et al.	0.07	0.38	34	0.22	0.5	34	4.9%	-0.33 [-0.81, 0.14]
Leenen et al.	31.7	4.6	45	30	5.4	41	5.2%	0.34 [-0.09, 0.76]
Markle-Reid et al.	8.27	1.57	75	8.05	1.45	64	5.6%	0.14 [-0.19, 0.48]
Miklavcic et al.	8.29	1.34	70	7.92	1.87	62	5.6%	0.23 [-0.11, 0.57]
Newhouse et al.	8.24	1.39	73	8.4	1.5	75	5.7%	-0.11 [-0.43, 0.21]
Nguyen et al.	7.6	1.4	68	6.17	1.47	67	5.5%	0.99 [0.63, 1.35]
Pack et al.	55.14	10.81	37	50	9.94	38	5.0%	0.49 [0.03, 0.95]
Park et al.	6.89	1.75	22	6.69	2.26	20	4.3%	0.10 [-0.51, 0.70]
Still et al.	46.62	10.07	30	45.48	13.92	30	4.8%	0.09 [-0.41, 0.60]
Wu et al.	220.5	20.44	67	127.46	61.9	45	4.9%	2.19 [1.71, 2.67]
Zeng et al.	57.41	6.34	22	53.05	4.03	22	4.2%	0.81 [0.19, 1.42]
**Subtotal (95% CI)**			**579**			**514**	**60.0%**	**0.46 [0.10, 0.82]**
Heterogeneity: Tau² = 0.35; Chi² = 90.97, df = 11 (P < 0.00001); I² = 88%								
Test for overall effect: Z = 2.48 (P = 0.01)								
**5.6.2 >6 months**								
Boer et al.	2.98	0.41	35	2.87	0.52	41	5.0%	0.23 [-0.22, 0.68]
Griva et al.	6.81	1.6	134	6.33	1.74	101	5.9%	0.29 [0.03, 0.55]
Jolly et al.	8.1	1.6	228	7.7	1.8	272	6.2%	0.23 [0.06, 0.41]
Rieger et al.	120.86	28.96	98	115.33	26.69	102	5.8%	0.20 [-0.08, 0.48]
Towfighi et al.	31.3	7.6	241	31.6	8.1	246	6.2%	-0.04 [-0.22, 0.14]
Wang1 et al.	30.21	3.9	39	24.48	2.67	39	4.7%	1.70 [1.18, 2.22]
Young et al.	3.97	0.91	158	3.95	0.97	161	6.1%	0.02 [-0.20, 0.24]
**Subtotal (95% CI)**			**933**			**962**	**40.0%**	**0.31 [0.05, 0.56]**
Heterogeneity: Tau² = 0.10; Chi² = 41.41, df = 6 (P < 0.00001); I² = 86%								
Test for overall effect: Z = 2.36 (P = 0.02)								
**Total (95% CI)**			**1512**			**1476**	**100.0%**	**0.41 [0.19, 0.62]**
Heterogeneity: Tau² = 0.19; Chi² = 141.18, df = 18 (P < 0.00001); I² = 87%								
Test for overall effect: Z = 3.66 (P = 0.0003)								
Test for subgroup differences: Chi² = 0.44, df = 1 (P = 0.51), I² = 0%								

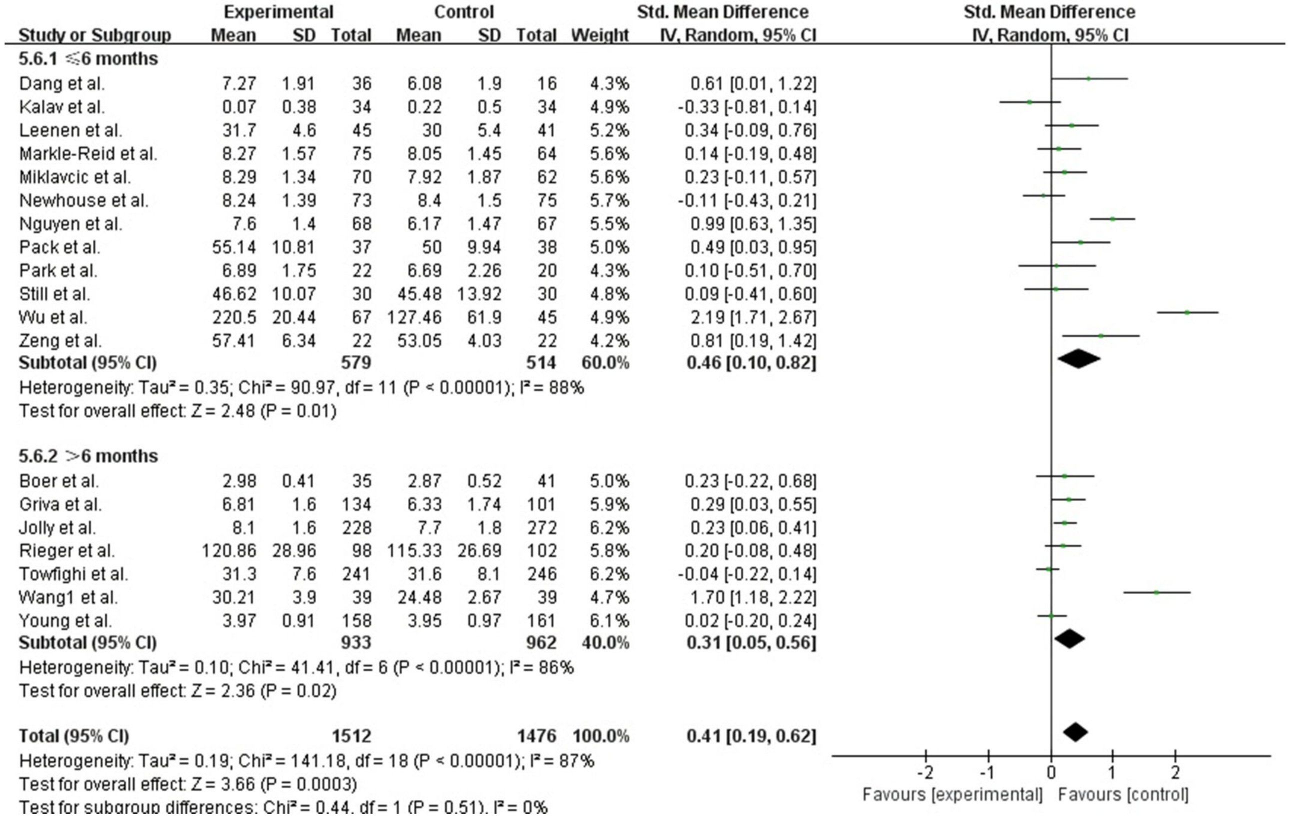

Figure S1. Forest plot: Subgroup analysis were performed based on mean age to explore its effect on Low-better QOL.

	Experimental			Control				Std. Mean Difference
Study or Subgroup	Mean	SD	Total	Mean	SD	Total	Weight	IV, Random, 95% CI
**1.14.1 <60**								
Dang et al.	42.83	27.03	36	44.88	26.41	16	6.8%	-0.08 [-0.66, 0.51]
Rieger et al.	59.9	19.06	98	65.2	18.03	102	9.9%	-0.28 [-0.56, -0.01]
**Subtotal (95% CI)**			**134**			**118**	**16.7%**	**-0.25 [-0.50, 0.01]**
Heterogeneity: Tau² = 0.00; Chi² = 0.40, df = 1 (P = 0.53); I² = 0%								
Test for overall effect: Z = 1.92 (P = 0.06)								
**1.14.2 ≥60**								
Aboumatar et al.	65.1	22	119	60.9	21	120	10.2%	0.19 [-0.06, 0.45]
Dalal et al.	24.1	20.9	92	27.5	23.2	93	9.8%	-0.15 [-0.44, 0.14]
Dorsch et al.	44.2	4	42	45.9	3.7	41	8.3%	-0.44 [-0.87, -0.00]
Farmer et al.	56.9	19.5	93	56.8	20.9	48	9.2%	0.00 [-0.34, 0.35]
Jiang et al	20.85	7.11	53	21.7	6.69	53	8.9%	-0.12 [-0.50, 0.26]
Rahimi et al.	34.7	26.4	86	34.5	25.9	87	9.7%	0.01 [-0.29, 0.31]
Wang1 et al.	18.3	3.5	39	20.9	4.4	39	8.1%	-0.65 [-1.10, -0.19]
Wang2 et al.	35.5	11.2	77	47.5	15.8	77	9.4%	-0.87 [-1.20, -0.54]
Zhang et al.	17.8	4.6	85	22	4.1	89	9.6%	-0.96 [-1.28, -0.65]
**Subtotal (95% CI)**			**686**			**647**	**83.3%**	**-0.32 [-0.61, -0.03]**
Heterogeneity: Tau² = 0.17; Chi² = 54.25, df = 8 (P < 0.00001); I² = 85%								
Test for overall effect: Z = 2.19 (P = 0.03)								
**Total (95% CI)**			**820**			**765**	**100.0%**	**-0.30 [-0.54, -0.06]**
Heterogeneity: Tau² = 0.13; Chi² = 54.68, df = 10 (P < 0.00001); I² = 82%								
Test for overall effect: Z = 2.46 (P = 0.01)								
Test for subgroup differences: Chi² = 0.15, df = 1 (P = 0.69), I² = 0%								

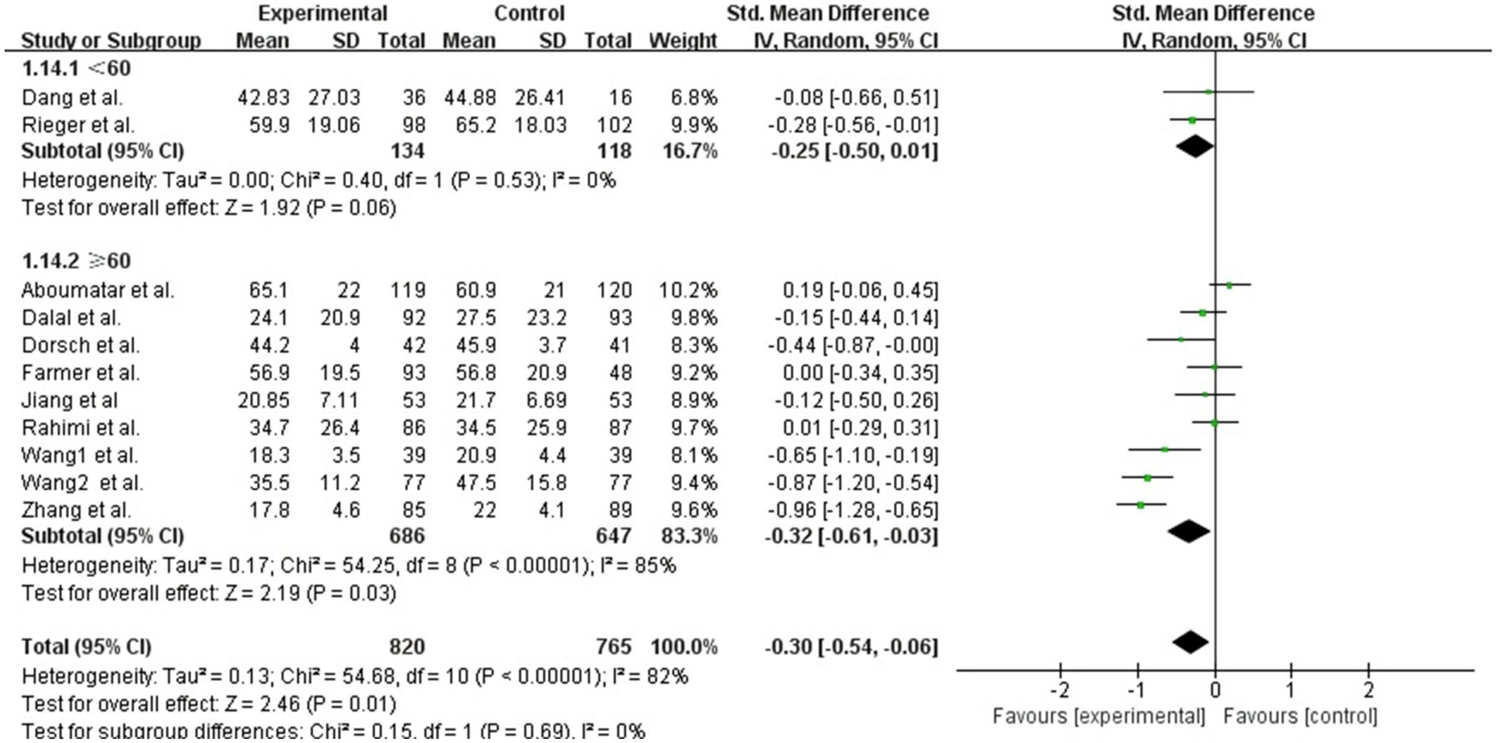

Figure S2. Forest plot: Subgroup analysis were performed based on follow-up times to explore its effect on self-efficacy.
